# Advanced materials and devices strategies of mechanical stimulation for tissue regeneration

**DOI:** 10.1093/rb/rbag054

**Published:** 2026-03-16

**Authors:** Mengting Huan, Huihui An, Xing Sheng, Lan Yin, Liu Wang

**Affiliations:** Key Laboratory of Biomechanics and Mechanobiology of Ministry of Education, Beijing Advanced Innovation Center for Biomedical Engineering, School of Biological Science and Medical Engineering, Beihang University, Beijing 100191, P. R. China; Key Laboratory of Biomechanics and Mechanobiology of Ministry of Education, Beijing Advanced Innovation Center for Biomedical Engineering, School of Biological Science and Medical Engineering, Beihang University, Beijing 100191, P. R. China; Department of Electronic Engineering, Beijing National Research Center for Information Science and Technology, Center for Flexible Electronics Technology, IDG/McGovern Institute for Brain Research, State Key Laboratory of Flexible Electronics Technology, Tsinghua University, Beijing 100084, P. R. China; School of Materials Science and Engineering, The Key Laboratory of Advanced Materials of Ministry of Education, State Key Laboratory of Flexible Electronics Technology, Tsinghua University, Beijing 100084, P. R. China; Key Laboratory of Biomechanics and Mechanobiology of Ministry of Education, Beijing Advanced Innovation Center for Biomedical Engineering, School of Biological Science and Medical Engineering, Beihang University, Beijing 100191, P. R. China

**Keywords:** mechanical stimulation, tissue regeneration, materials, devices

## Abstract

Mechanical stimulation as a form of physical therapy plays a crucial role in regulating cellular behavior and promoting tissue regeneration. It can influence cell proliferation, differentiation, migration and extracellular matrix deposition, thereby providing a powerful biophysical cue in regenerative medicine. The implementation of precisely controllable mechanical stimulation relies on efficient and reliable tool platforms that can deliver defined magnitudes, frequencies, directions and temporal patterns of force, while maintaining high reproducibility and safety in biological environments. For mechanical stimulation of cells and injured tissues, a variety of materials as well as devices with distinct force outputs, such as stretchable elastic substrates, microfluidic shear systems and mechano-active scaffolds have been designed to meet diverse experimental and therapeutic requirements. Moreover, functional materials integrated with magnetic, acoustic and optical modalities are also been developed to establish remote mechanical stimulation systems, enabling spatiotemporally programmable interventions even in deep or delicate tissues. This review summarizes recent advances of the *in vitro* and *in vivo* strategies that leverage mechanical stimulation in regenerative medicine, along with its regulatory effects on regenerative processes in neural, skeletal, muscular and other tissues. Finally, the major challenges and prospects regarding materials and devices for mechanical stimulation in tissue therapy are discussed.

## Graphical abstract

**Graphical Abstract:**
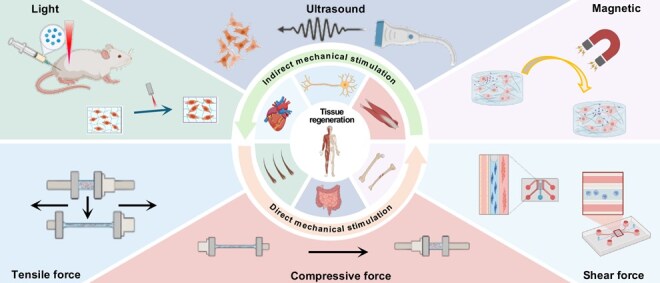

## Introduction

Among numerous biophysical cues, mechanical stimulation has garnered widespread attention due to its conservation across all stages of life [1–4]. It plays an indispensable role in regulating cellular behaviors as well as maintaining tissue and organ functions throughout an organism’s lifespan [5–13]. Notably, mechanical stimulation has a long history of clinical application. As a traditional form of mechanical stimulation, massage therapy has been widely used to relieve muscle strain, promote local blood circulation and accelerate soft tissue repair [14–17]. As a therapeutic modality, mechanical stimulation has shown considerable potential in promoting tissue regeneration and functional recovery such as nerve, muscle and bone [18–25]. This potential is also manifested in modern commercial devices, such as the commonly available fascia gun on the market. By generating mechanical stimulation through high-frequency vibration, it can penetrate deep muscle tissue, relieve muscle spasms and improve muscle metabolism [26–28]. Meanwhile, emerging evidence indicates that mechanical stimulation promotes tissue regeneration by mimicking physiological conditions *in vivo* through regulating diverse cellular processes such as adhesion [29, 30], migration [31, 32] and proliferation [33, 34]. In addition, mechanosensitive cues directly modulate extracellular matrix assembly, composition and architectural organization, thereby exerting profound influences on cell-matrix interactions [35–38].

The implementation of precisely controllable mechanical stimulation relies on efficient and reliable tool platforms. Currently, *in vitro* force-stimulation systems can be broadly categorized into three types [39]. The first approach involves modulating the mechanical properties of cell culture substrates, often by using matrices with tunable stiffness, such as hydrogels [40–43]. Studies have shown that substrate rigidity plays a critical role in regulating diverse cellular processes such as adhesion [29], migration [31], proliferation [33] and differentiation [44]. The second category employs topographic cues on substrate surfaces to transmit mechanical signals [45]. Nanopatterned substrates have been shown to promote highly aligned collagen fiber formation by guiding fibroblasts, osteoblasts and tenocytes [46–48]. Nevertheless, a major limitation of this strategy is the static nature of substrates, which lack dynamic tunability. The third approach involves the direct application of diverse force types onto cells or tissues. Generally, bioreactors that apply low-strain periodic axonal stretching to cells are adopted [49–51]. However, these devices are highly bulky and complex, hindering their translation from *in vitr*o models to *in vivo* clinical applications.

To more realistically reproduce physiological conditions, researchers have also developed various *in vivo* force stimulation systems. Various customized external traction devices are designed to perform daily progressive extensions of the nerve. The advantage of these methods is its ability to achieve dynamic stretch regulation, simulating the repetitive dynamic strains that cells experience in their *in vivo* environment and providing a dynamic mechanical stimulation environment for the cells. However, it is limited by cumbersome operation, invasiveness and susceptibility to infection [18, 52, 53].

To overcome the constraints of invasive procedures, remote manipulation systems based on magnetic materials, light-responsive materials and ultrasound-responsive materials exhibit distinct advantages, including a reduced risk of infection and complications, as well as precisely control the stimulation site and intensity [3, 54–60]. While these approaches overcome spatial constraints inherent in *in vitro* models, it confronts numerous engineering challenges, including maintaining long-term stability of biocompatible materials and managing foreign body responses elicited by implanted devices, as well as the nondegradability requiring a secondary surgical procedure for removal [61–65].

**Figure 1:**
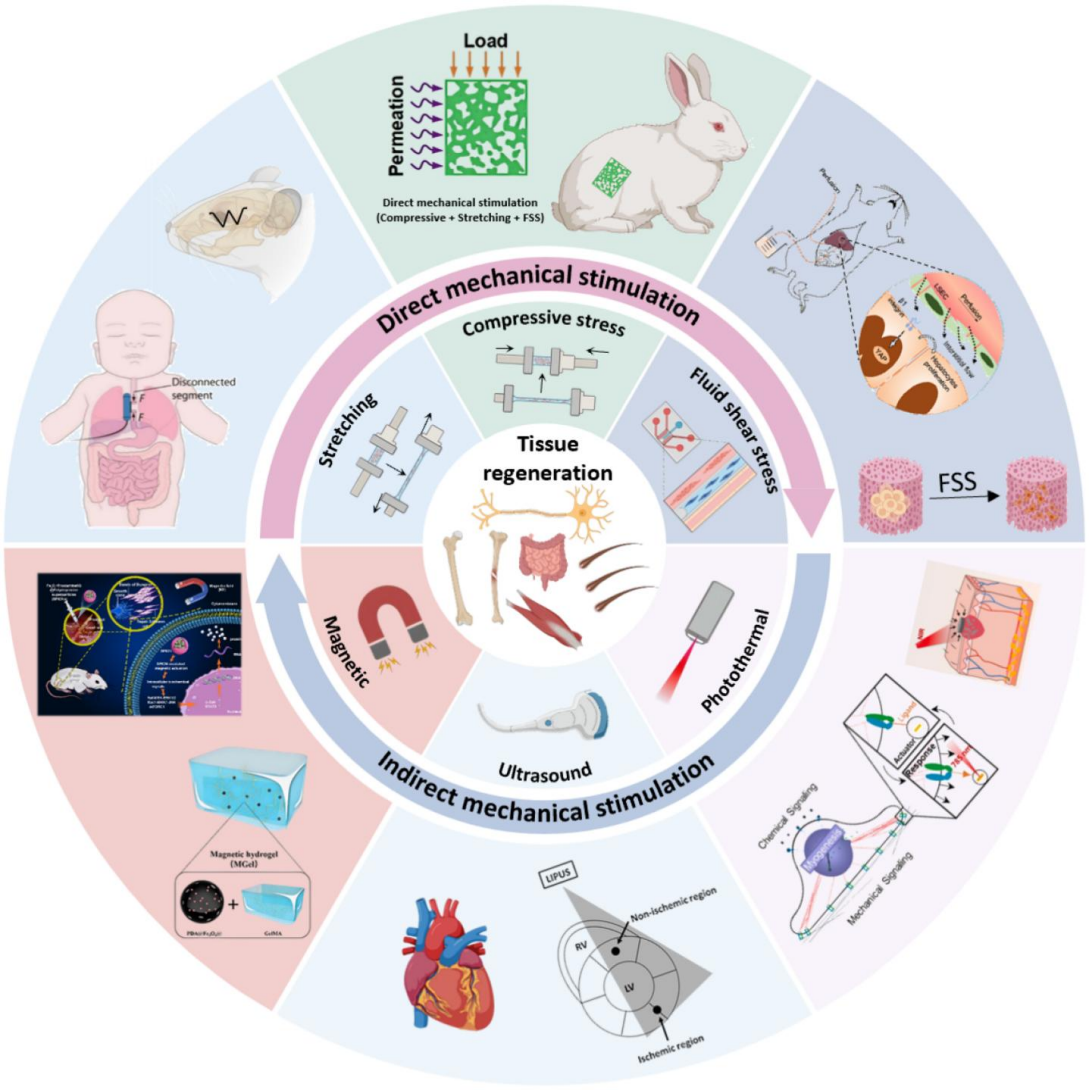
*In vitro* and *in vivo* mechanical stimulation strategies for various tissue regeneration. Created with BioRender.com.

In summary, mechanical stimulation is critically involved in the development, remodeling and maintenance of tissues such as nerves, muscles and bones. This review summarizes the current understanding of how mechanical stimulation promotes cell proliferation, differentiation and tissue regeneration. We systematically study the applied strategies, both *in vitro* and *in vivo*, that leverageing mechanical stimulation in regenerative medicine, along with its effects on regenerative processes in neural, skeletal, muscular and other tissues (Figure 1). Finally, we discuss the major challenges and prospects of the materials and devices for mechanical stimulation in tissue therapy.

## Mechanical stimulation promotes nerve regeneration

Research indicates that organismal growth and development can be regarded as a form of passive mechanical stimulation [66]. Since the 1940s, mechanical stimulation has been recognized as a physical intervention capable of promoting axonal elongation and exerting significant effects on neural regeneration [66]. Subsequent studies reveal that although sensory axons regenerate at a rate of approximately 1 mm/day after injury, the application of mechanical stimulation to stretch axonal growth cones can accelerate this process, achieving maximum regenerative rates of up to 8 mm/day [67]. Notably, even after extending 10 cm in length, the cytoskeletal architecture remains largely intact. In addition, studies also demonstrate that appropriate mechanical stimulation of the peripheral nerve could accelerate Schwann cells (SCs) proliferation, enhance reparative SCs effects on axons and significantly elongate nerve tissue within a given timeframe, thereby enhancing neural regeneration [68]. Although the precise mechanisms by which mechanical stimulation facilitates regeneration remain incompletely understood, this regeneration effect is believed to stem from its ability to activate mechanosensitive channels such as PIEZO and transient receptor potential vanilloid (TRPV) [69–74].

### *In vitro* mechanical stimulation materials and devices for nerve regeneration

Mechanical forces play a pivotal role in regulating neuronal behavior and maintaining nervous system function [66, 75]. The dynamic interplay between nerve cells and their microenvironment encompasses not only biomolecular interactions but also extracellular matrix connections and mechanical forces, all of which are critical for neural regeneration [76, 77]. For instance, during the process of nerve injury repair, processes including axonal growth, cell differentiation, assembly, formation of higher-order structures and morphogenetic events during embryonic development rely on mechanical force regulation [78, 79]. *In vitro* studies have demonstrated that controlling the morphology of nerve cells can directionally induce their transformation into a repair phenotype [68, 80]. Therefore, employing mechanical stimulation to promote nerve regeneration holds significant scientific relevance and translational potential.

**Figure 2:**
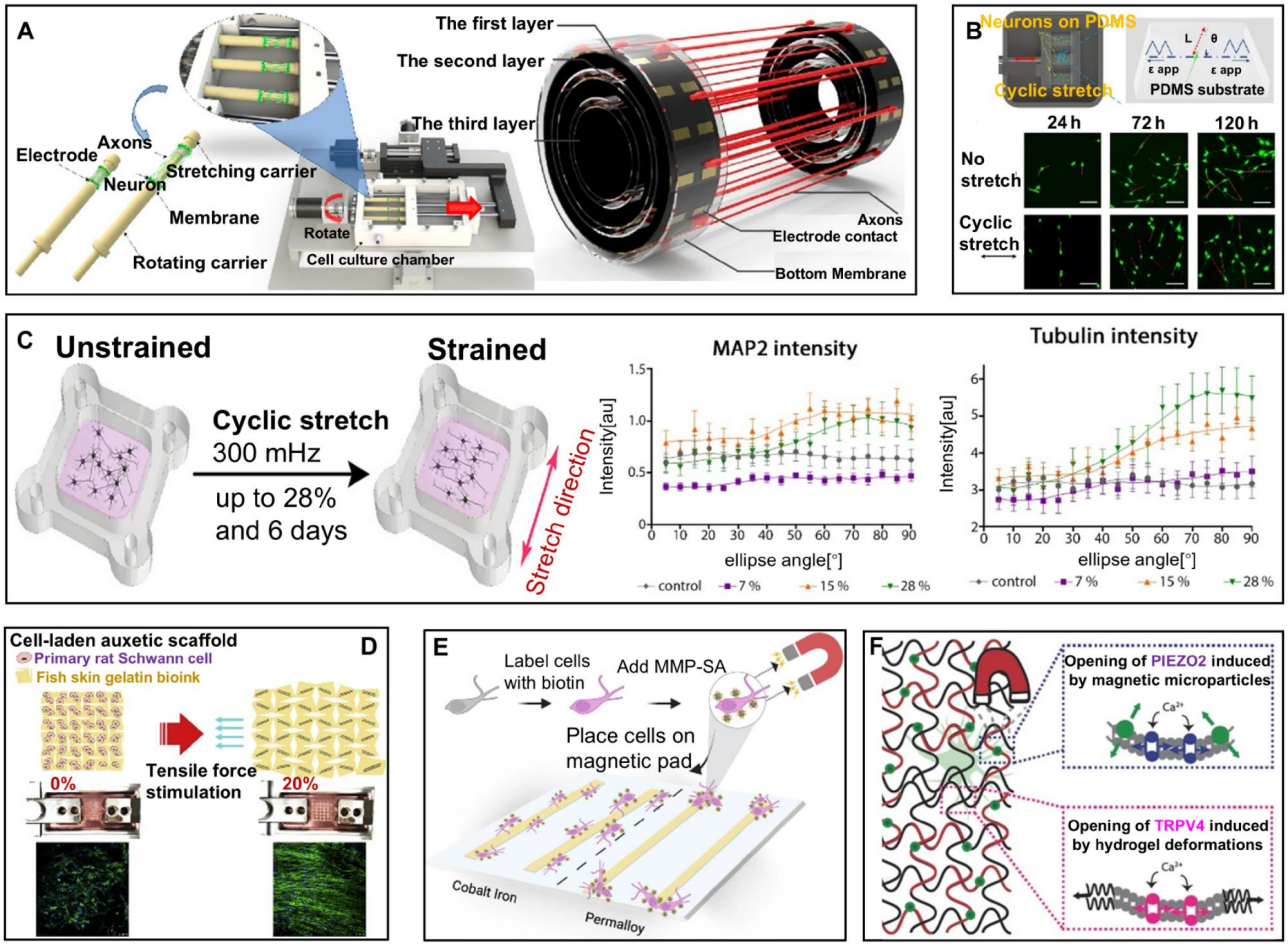
*In vitro* devices for nerve repair and regeneration. (**A**) A 3D bioengineered device applying tensile forces to drive neuronal growth. Reproduced from Ref. [81] with permission of Multidisciplinary Digital Publishing Institute, © 2022. (**B**) A cyclic stretching device facilitates neuronal reorientation and promotes axonal growth. Reproduced from Ref. [82] with permission of Frontiers Media S.A., © 2020. (**C**) An *ex vivo* system designed to evaluate neuronal responses to periodic strain functioning as a mechanical signal. Reproduced from Ref. [83] with permission of American Chemical Society, © 2018. (**D**) Periodic stretching of hydrogel scaffolds applies tensile stimulation to SCs. Reproduced from Ref. [84] with permission of Elsevier, © 2020. (**E**) A magnetic system based on the streptavidin-biotin interaction, which is colloidal magnetic particles covalently coupled with a monolayer of streptavidin. Reproduced from Ref. [85] with permission of Springer Nature, © 2023. (**F**) Mechanical stimulation of DRG neurons by a 3D hyaluronic acid hydrogel incorporating magnetic microparticles activates the mechanically sensitive PIEZO2 channels and TRPV4 channels. Reproduced from Ref. [86] with permission of Wiley-VCH, © 2018.

To investigate the effect of mechanical stimulation on neural cells, Li *et al*. [81] developed a three-dimensional (3D) neural traction growth device. This system enables both rotational and linear movement modes. By using a culture medium based on a circle as the carrier, its uniform rotation ensures the even attachment of neural cells onto the membrane, preventing cell aggregation. Meanwhile, linear motion facilitates the stretching of axons. The membrane connected to the traction block moves, thereby pulling the nerve cells attached to it, achieving the stretching of the nerve cell axons (Figure 2A). In addition, Lin *et al*. [82] developed a device that applies cyclic stretch to neurons by deforming a polydimethylsiloxane (PDMS) substrate, with strains ranging from 2% to 10% and frequencies between 0.05 Hz and 0.25 Hz. They observed that when subjected to cyclic stretching at an amplitude of 10% and frequency of 0.25 Hz, PC12 cells progressively realigned their orientation perpendicular to the direction of strain. (Figure 2B). Besides, Abraham *et al*. [83] also constructed an experimental system capable of applying periodic stretch strain to neurons (Figure 2C). The system uses cross-linked PDMS as a flexible cell culture substrate material and achieves the stretching function through precise control of PDMS. The cyclic stretch experiment is carried out 24 h after neuron culture to allow cells to recover and adhere. Uniaxial strain with different amplitudes (7%, 15% or 28%) and a repeat frequency of 300 mHz is applied, thereby realizing the periodic stretching of neurons. This system not only significantly enhanced neuronal growth rates but also effectively promoted the formation of neuronal branch structures. Notably, microtubule-associated protein 2 (MAP2) and tubulin, established markers of neuronal differentiation, showed upregulated expression levels.

To further accelerate nerve regeneration, Chen *et al*. [84] demonstrated a dual-regulation strategy, combining mechanical stimulation with nerve growth factor (NGF) produced by mechanical stimulation. Using gelatin methacryloyl (GelMa)-based bioink as a carrier, human stem cells (hSCs) were precisely positioned within specially designed auxetic scaffold systems via 3D bioprinting technology. The scaffold has a negative Poisson’s ratio, which enables it to transfer compressive loads isotropically to the cells. Moreover, it can withstand a tensile strain of up to 20% without tearing (Figure 2D). During cell cultivation, the application of dynamic mechanical tension significantly enhanced cellular proliferation activity and differentiation efficiency, while simultaneously inducing the transcription and expression of NGF. Notably, NGF not only possesses pro-regenerative functionality but also exerts synergistic effects with mechanical stretching, effectively guiding cellular alignment and accelerating regenerative processes.

Moreover, to develop noninvasive neural modulation technologies, mechanical stimulation is employed in conjunction with magnetic forces and various magnetic materials [87]. For instance, a streptavidin-biotin interaction-based magnetic system (Figure 2E), which is colloidal magnetic particles covalently coupled with a monolayer of streptavidin (MMP-SA). Magnetic forces are exerted on the cell membranes bound to the particles, promoting migration along the micromagnet orientation and thereby enhancing tissue and cellular growth [85]. Furthermore, Tay *et al*. [86] described a 3D magnetic hyaluronic acid hydrogel (Figure 2F) that enables noninvasive neural modulation through magnetomechanical stimulation of dorsal root ganglion (DRG). This hydrogel facilitates healthy functional neurite growth and expression of excitatory and inhibitory ion channels. The results show that acute magnetomechanical stimulation primarily induces calcium influx in DRG via endogenous mechanosensitive TRPV4 and PIEZO2 channels. Chronic magnetomechanical stimulation, however, reduces PIEZO2 channel expression, offering a strategy to regulate mechanically sensitive channels that are typically overexpressed in pain conditions.

### *In vivo* mechanical stimulation materials and devices for nerve regeneration

**Figure 3:**
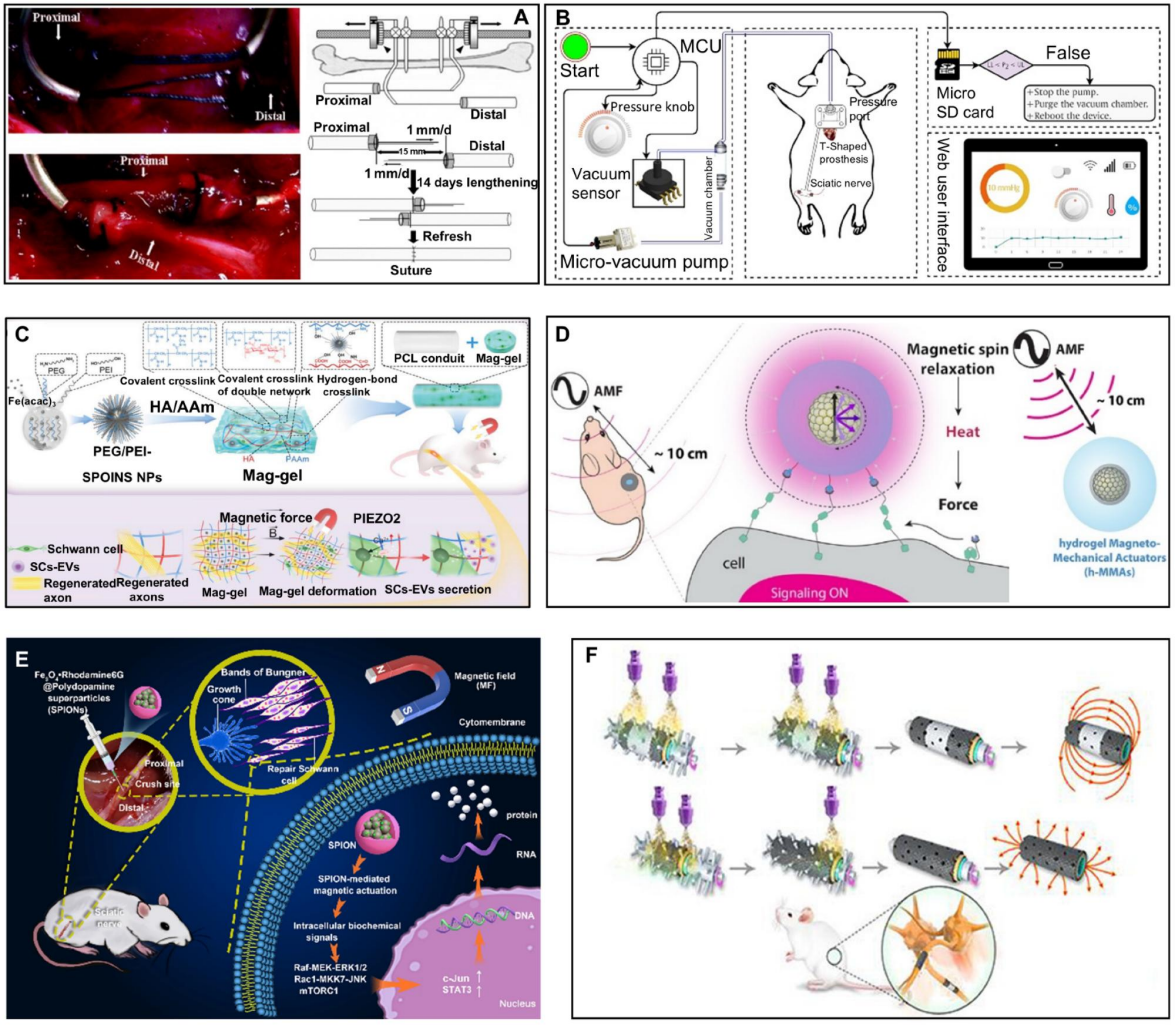
Mechanical force stimulus therapy devices for nerve regeneration *in vivo*. (**A**) A neural elongation device simultaneously gradually extends proximal and distal nerve stumps to repair peripheral nerve defects in rats. Reproduced from Ref. [52] with permission of John Wiley and Sons Inc., © 2008. (**B**) A T-shaped neural prostheses to stretch the nerve stump *in vivo*. Reproduced from Ref. [18] with permission of Elsevier, © 2020. (**C**) A superparamagnetic nanocomposite scaffold capable of controlling extracellular vesicle production on demand. Reproduced from Ref. [93] with permission of Elsevier, © 2023. (**D**) Schematic illustration of the hMMA nanoparticle, converting magnetic anisotropic energy into mechanical energy to activate target mechanical receptors. Reproduced from Ref. [59] with permission of BioMed Central, © 2022. (**E**) Schematic diagram of the mechanism by which SPIONs promote sciatic nerve regeneration and functional recovery. Reproduced from Ref. [58] with permission of American Chemical Society, © 2023. (**F**) A novel superparamagnetic multifunctional nerve scaffold. Reproduced from Ref. [57] with permission of Society for Neuroscience, © 2020.

Although various devices capable of delivering mechanical forces to cells have been designed and developed, which has greatly advanced the understanding of biological responses to mechanical stimulation [88–91]. Many of these devices are bulky and complex, hindering their applicability from *in vitro* experiments to *in vivo* applications. Therefore, *in vivo* mechanical stimulation strategies attracted numerous interests [92]. For example, Saijilafu *et al*. [52] designed an externally driven mechanical traction device (Figure 3A) to gradually stretch the proximal and distal stumps of injured rat sciatic nerves. Two stainless steel half-pins were vertically inserted into the femur of the rat, and an external nerve elongation device was mounted on these pins. The traction sutures attached to the nerve stumps were passed through a thin stainless-steel tube. Gradual nerve elongation was achieved by applying traction via a pulling ring at a rate of 1 mm per day to both the proximal and distal stumps of the injured sciatic nerve in rats. Specifically, after 14 days of continuous traction-induced lengthening, the two stumps of a 15 mm sciatic nerve defect were overlapped. However, the external fixators carry a significant risk of infection by preventing complete wound closure during traction.

To overcome the aforementioned potential risk of infection associated with external auxiliary devices. Sana Ullah Sahar *et al*. [53] proposed a method that utilizes the controllable vacuum volume inside T-shaped neural prostheses to stretch the proximal nerve stump. Furthermore, to refine the design and optimize the performance of neural scaffolds, they also developed a device that applies directly controlled axial tension to peripheral nerves *in vivo*, as shown in Figure 3B [18]. The electronic circuitry of this device comprises a microcontroller, vacuum sensor, solenoid valve and miniature vacuum pump. By integrating state-of-the-art online vacuum monitoring capabilities, it enables real-time control, data collection, processing and visualization. This apparatus serves as a tool to enhance the rate of peripheral nerve growth associated with mechanical stretching. Additionally, it can be employed to investigate mechanotransduction mechanisms in peripheral nerves *in vivo*.

Additionally, to apply mechanical stimuli in a noninvasive manner, Xia *et al*. [93] have constructed a superparamagnetic nanocomposite scaffold capable of controlling extracellular vesicle production on demand (Figure 3C). This scaffold is highly sensitive to rotating magnetic fields and can serve as a mechanical stimulation platform to apply microscale/nanoscale forces to encapsulated SCs, thereby regulating the production of SCs-derived extracellular vesicles.

Furthermore, to achieve deep penetration of input signals and efficient force generation, Jeong *et al*. [59] introduced a hydrogel magnetomechanical actuator (hMMA) nanoparticles (Figure 3D) for wireless, targeted and long-range stimulation *in vivo*. These hMMA nanoparticles efficiently convert magnetic anisotropy energy into mechanical work through a two-step process involving magneto-thermal followed by thermo-mechanical energy transduction. This induces contraction of the surrounding polymer shell, which applies mechanical tensile stress to target proteins under oscillating magnetic field excitation. Mechanosensitive proteins transmit signals by unfolding force-sensitive domains upon mechanical traction, thereby activating target mechanoreceptors. Notably, the designed hMMA nanoparticles serve as a potent *in vivo* perturbation tool, enabling mechanobiological investigations in a minimally invasive and unconstrained manner.

Similarly, Liu *et al*. [58] designed and fabricated fluorescent-magnetic dual-functional superparamagnetic iron oxide nanoparticles (SPIONs) (Figure 3E). By establishing a rat sciatic nerve injury model, SPIONs were applied to SCs, and a targeted magnetic actuation system for the sciatic nerve was developed. Interacting with an external magnetic field, SPIONs initiated mechanotransduction processes. SCs sensed these exogenous magnetically driven mechanical forces and converted them into intracellular biochemical signals. Through modulating the expression of regeneration-associated genes, SPIONs induced and sustained a reparative phenotype in SCs, ultimately promoting sciatic nerve regeneration and functional recovery.

To eliminate the inconvenience of carrying external stimulators, Qian *et al*. [57] employed a 3D multilayer approach to design a gradient magnetized iron oxide nanoparticle scaffold (GIONS) (Figure 3F). Compared to the relatively random arrangement of hyaluronic acid-decorated superparamagnetic iron oxide nanoparticles (HIONS), the design of GIONS eliminates the need for complex magnetic field regulation. Instead, through a fabrication method combining regional selective deposition and multilayer structure fixation, it precisely controls ferrosoferric oxide (Fe_3_O_4_) nanoparticles to deposit only in the proximal one-third region and distal one-third region of the scaffold, leaving the middle one-third region uncoated. This configuration naturally forms a gradient magnetic field with intensity gradually decreasing from both ends toward the center. The design not only reduces the total dosage of nanoparticles to mitigate potential toxicity but also provides directional physical cues for cells via the gradient magnetic field, ultimately achieving directed neurite outgrowth. The polycaprolactone scaffold loaded with Fe_3_O_4_ nanoparticles produces mechanical signals through a self-magnetization mode, further promoting *in vivo* activation of glial cells and immunomodulation of macrophages. This enhances nerve conduction velocity and motor function, thereby accelerating multifunctional regeneration in cases of severe tissue injury.

## Mechanical stimulation promotes bone repair

In recent years, studies have demonstrated that mechanical stimulation plays a crucial role in bone formation and remodeling [94]. Bone tissue not only responds passively but can also actively sense and convert external mechanical signals, participating in bone remodeling [95]. In the skeletal system, osteoblasts are one of the main cell types responsible for maintaining bone homeostasis. They originate from the differentiation of mesenchymal stem cells (MSCs) and possess the abilities of differentiation, proliferation and apoptosis, making them crucial in bone formation [96, 97]. Their biological functions are regulated by hormones, cytokines and mechanical forces. Mechanical forces can modulate the proliferation, differentiation and apoptosis of osteoblasts by altering cell morphology, signaling pathways and gene expression [98, 99]. Additionally, mechanical signals can be transformed into intracellular biochemical signals via mechanoreceptors such as integrin receptors, focal adhesions and cadherins, thereby activating pathways including Wnt/β-catenin and receptor activator of nuclear factor-κB ligand/receptor activator of nuclear factor-κB/osteoprotegerin (RANKL/RANK/OPG) and exerting effects through a series of enzymatic reactions [98, 100–102]. These pathways interact to form a regulatory network that precisely controls osteoblast function at different stages [98]. Furthermore, mechanical stimulation influences the morphology and structure of osteoblasts, promotes the formation of fibrillar connections, regulates the synthesis and alignment of extracellular matrix (ECM), and thereby improves the structure and function of bone tissue. Morphological changes can further enhance cellular sensitivity to subsequent mechanical signals, forming a positive feedback loop that maintains the dynamic balance and stability of bone tissue [103].

### *In vitro* strategies of materials and devices for mechanical stimulation promoting bone repair

*In vitro* studies have widely employed mechanical stimulation to investigate its mechanisms and application potential in bone repair. Various forms of stimulation, including fluid shear stress (FSS), cyclic tensile strain and magneto-mechanical cues have been shown to regulate the proliferation and differentiation of osteoblasts and MSCs [104–107].

**Figure 4:**
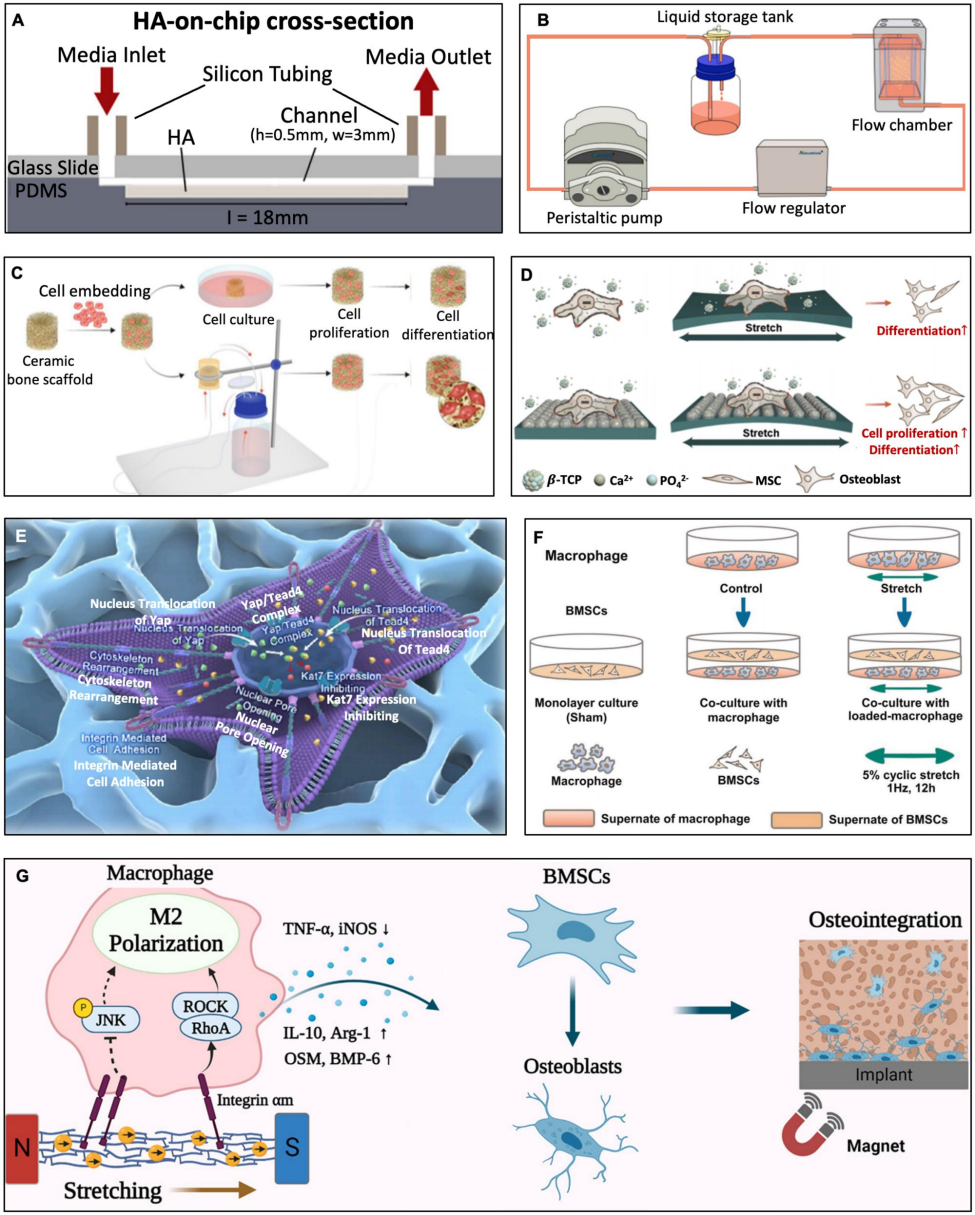
*In vitro* strategies on the promotion of bone repair. (**A**) Schematic cross-section of the HA-on-chip. Reproduced from Ref. [109] with permission of Elsevier, © 2021. (**B**) Applying equipment of fluid shear stress. Reproduced from Ref. [110] with permission of Elsevier, © 2022. (**C**) TiO_2_ scaffolds in a perfusion flow bioreactor. Reproduced from Ref. [111] with permission of MDPI, © 2022. (**D**) Neoteric semiembedded β-tricalcium phosphate promotes osteogenic differentiation of mesenchymal stem cells under cyclic stretch. Reproduced from Ref. [114] with permission of American Chemical Society, © 2024. (**E**) Hydrogel enhanced organoid multidirectional differentiation via Yap/Tead4 mechanotransduction for accelerated tissue regeneration. Reproduced from Ref. [113] with permission of American Chemical Society, © 2025. (**F**) Coculture with stretched macrophages promotes osteogenic differentiation of BMSCs. Reproduced from Ref. [112] with permission of Wiley, © 2024. (**G**) Remote activation of M2 macrophage polarization via magneto-mechanical stimulation to promote osteointegration. Reproduced from Ref. [115] with permission of American Chemical Society, © 2023.

FSS is a type of mechanical stimulation caused by the flow of extracellular fluid across the cell membrane surface [108]. The application of load to the skeleton induces interstitial fluid movement, which subsequently compresses the lacunar-canalicular system (LCS) and triggers a variety of mechanical stimulations. Atif *et al*. [109] integrated calcium-deficient hydroxyapatite (HA) into a microfluidics-based platform (HA-on-chip), where medium flow was generated through microfluidics technology to mimic the fluid flow and shear stress environment in bone tissue under mechanical loading (Figure 4A). They found that at a flow rate of 8 μL/min, the mouse calvaria derived cell line (MC3T3-E1) on the HA-on-chip exhibited significantly higher proliferation capacity. Moreover, Guo *et al*. [110] constructed a custom fluid shear-stress loading device to impose continuous laminar shear stress on cells (Figure 4B). The culture medium was recirculated from the flow chamber back to the reservoir via a peristaltic pump, stabilizer and flow chamber, so that cells inside the chamber were continuously exposed to fluid and could sense FSS. They inserted the cell-seeded glass slides, oriented perpendicular to the horizontal plane of the flow chamber, into four parallel channels to apply FSS to bone marrow mesenchymal stem cells (BMSCs). The results showed that exposing BMSCs to 4 dyne/cm^2^ for 2 h each day over 3 days significantly promoted osteogenic differentiation. Besides, the effects of FSS also varied depending on scaffolds and culture conditions. Schröder *et al*. [111] while studying osteoblast culture on titanium dioxide (TiO_2_) scaffolds in a perfusion-flow bioreactor, found that dynamic culture significantly improved cell distribution within scaffolds and increased cell numbers by 5-fold after 21 days (Figure 4C). At a flow rate of 0.08 mL/min, osteopontin (OPN) mRNA expression in MC3T3-E1 cells was enhanced 40-fold at Days 7 and 21, respectively, while collagen type I alpha I expression increased 18-fold at Day 21; in contrast, a 0.16 mL/min flow rate was less effective. These findings indicate that different FSS patterns and intensities elicit varied osteoblast responses. Thus, factors such as shear stress magnitude, direction and duration must be comprehensively considered in related research.

In addition, tensile stress is also an important form of mechanical stimulation affecting osteoblast function [116]. Appropriate tensile stress can promote osteoblast proliferation, differentiation and bone matrix synthesis. For example, Dai *et al*. [114] reported a synergistic strategy of integrating biomaterials with mechanical stimulation by engineering an elastic polymer membrane that semi-embeds β-tricalcium phosphate (β-TCP) nanoparticles to culture MSCs under cyclic stretching condition. Upon uniaxial cyclic stretching (UCS; 10% strain, 1 Hz), the scaffold significantly up-regulated osteogenic gene expression in MSCs (Figure 4D). Mechanistic studies have demonstrated that this effect was closely related to activation of the PIEZO1 signaling pathway by uniaxial cyclic stretch, effectively promoting osteogenic differentiation of MSCs. Additionally, some studies have focused on providing spatial mechanical stimulation directly through scaffold surface microenvironments. Luo *et al*. [113] developed a GelMA-based biomimetic hydrogel that induced cytoskeletal rearrangement through cell adhesion, thereby delivering spatial mechanical stimulation (Figure 4E). This significantly enhanced the multilineage differentiation potential of BMSCs, accelerating regeneration of bone, cartilage and tendon. The mechanism was closely related to Lysine acetyltransferase (KAT7) downregulation mediated by the Yes-associated protein (YAP)/TEA domain transcription factor 4 (TEAD4) mechanotransduction pathway, thereby promoting osteogenic, chondrogenic and tenogenic differentiation of BMSCs.

Moreover, mechanical stimulation can promote bone regeneration not only by directly acting on osteoblasts but also indirectly through immunocyte modulation [117]. Dong *et al*. [112] co-cultured BMSCs with macrophages subjected to 5% cyclic mechanical stretch (1.0 Hz, 12 h). They observed that BMSCs exposed to stretched macrophages adopted a highly branched ‘osteocyte-like’ morphology displaying long filopodia and thick stress-fiber formation, whereas BMSCs co-cultured with unstretched macrophages or control cells retained a polygonal shape with fewer branches and thin actin filaments (Figure 4F). The underlying mechanism is that cyclic mechanical stretch induced macrophage polarization toward the M2 phenotype, leading to the secretion of anti-inflammatory factors such as interleukin-10 (IL-10) and transforming growth factor-β (TGF-β), thereby modulating the inflammatory microenvironment. This also activated YAP and promoted its nuclear translocation, upregulating bone morphogenetic protein 2 (BMP2) expression, thus significantly enhancing BMSC osteogenic differentiation capacity. Furthermore, Shao *et al*. [115] developed a novel magnetically responsive mineralized collagen coating with superparamagnetic iron oxide nanoparticles (MC + IOPs) capable of inducing remote magneto-mechanical stimulation of macrophages under an external magnetic field (Figure 4G). This stimulation induced M2 polarization of macrophages, optimized the inflammatory microenvironment by triggering integrin-related cascades and inhibiting c-Jun N-terminal kinase (JNK) phosphorylation in the mitogen-activated protein kinase (MAPK) pathway, and thereby promoted osteogenic differentiation of BMSCs and *in vivo* osseointegration. *In vitro*, conditioned medium from magneto-mechanically stimulated macrophages significantly enhanced osteogenesis-related gene expression, Alkaline phosphatase (ALP) activity and mineralized matrix deposition.

### *In vivo* and clinical biomaterial-based strategies on mechanical stimulation promoting bone repair

Animal models and patient treatments have demonstrated that mechanical forces can not only accelerate bone formation but also regulate the inflammatory-immune-bone axis, contributing to favorable healing outcomes [98, 118, 119]. Approaches such as distraction osteogenesis, mechanical loading and intelligent scaffold designs reveal that mechanical cues enhance mesenchymal stem cell recruitment, macrophage polarization and angiogenesis, ultimately supporting robust bone remodeling [103, 120, 121]. These findings also indicate that translating precise mechanical parameters from laboratory to clinical practice is vital for maximizing therapeutic benefits [122]. Moreover, advanced biomaterial-based systems integrating mechanical responsiveness, such as magneto-mechanical scaffolds or porous architectures, illustrate the potential of combining mechanical stimulation with other regenerative strategies for optimized *in vivo* bone repair [115, 123].

**Figure 5:**
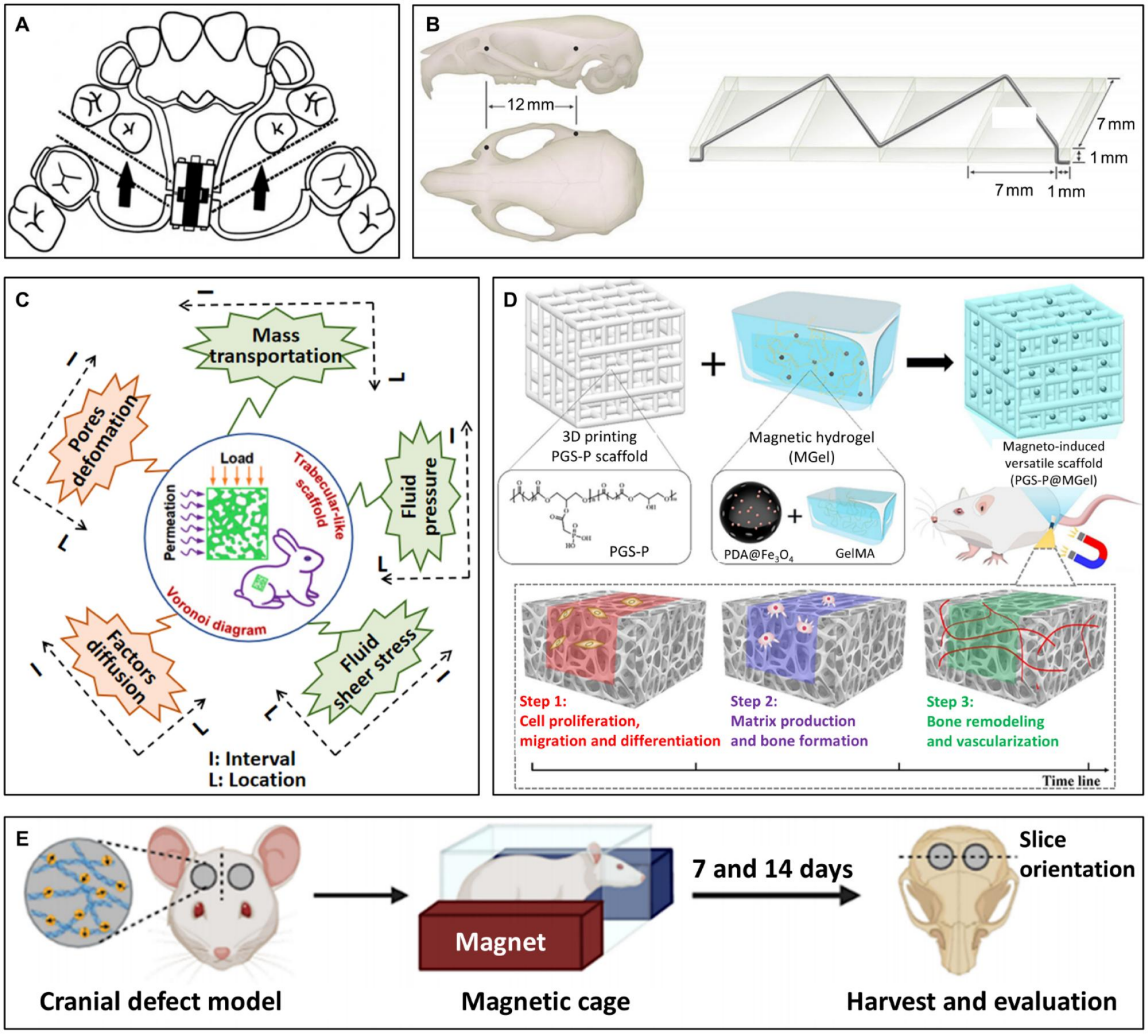
*In vivo* biomaterial-based and clinical strategies on the promotion of bone repair. (**A**) The clinical diagrams for the procedure of MASDO. Reproduced from Ref. [124] with permission of Springer Nature, © 2024. (**B**) Schematic illustration of the holes made on the zygomatic and maxilla bones to place the expansion appliance, and an illustration of the expansion appliance. Reproduced from Ref. [125] with permission of Springer Nature, © 2024. (**C**) The mechanical stimulation environment with diversification in location and interval formed in the trabecular-like structure. Reproduced from Ref. [126] with permission of Elsevier, © 2022. (**D**) Schematic illustration of the preparation procedures of PGS-P@MGel and the application process. Reproduced from Ref. [127] with permission of Wiley, © 2025. (**E**) Magneto-mechanical stimulation modulated M2 polarization of macrophages in the rat cranial defect model. Reproduced from Ref. [115] with permission of American Chemical Society, © 2023.

Clinically, maxillary anterior segmental distraction osteogenesis (MASDO) has been proposed as an effective technique for lengthening the maxilla (Figure 5A). This method creates a distraction segment in the anterior maxilla, advancing it forward while preserving posterior maxillary structures. Since the distractor is placed intraorally, MASDO not only lengthens the maxilla but also helps align crowded premolars, as the distraction segment is mainly in this region. Moreover, posterior maxillary position and velopharyngeal volume remain unchanged, avoiding adverse effects on velopharyngeal insufficiency function [124].

In addition, Liang *et al*. [125] established a trans-sutural distraction osteogenesis (TSDO) rat model and confirmed that mechanical stretching could promote bone regeneration at the osteogenic front of the zygomaticomaxillary suture, thereby achieving midfacial bone advancement. They implanted a W-shaped nickel-titanium alloy distractor in the experimental group (Figure 5B) with an initial applied force of 50 g. After 14 days of stretching, it was found that the experimental group exhibited more new bone formation, higher bone mineral density and greater bone mass. Subsequently, they further revealed the close association between mechanical stretching and immunoregulation in bone regeneration at the mechanism level. *In vitro* experiments showed that sinusoidal stretching with 10% intensity and 0.5 Hz frequency for 48 h could significantly enhance M2 macrophage polarization, promote the osteogenic differentiation of suture-derived stem cells and improve the angiogenic capacity of human umbilical vein endothelial cells.

Beyond direct distraction osteogenesis, strategies combining material engineering with mechanical stimulation also show great promise in promoting bone regeneration. Liang *et al*. [126] designed a Ti-6Al-4V porous scaffold with a trabecula-like architecture based on Voronoi diagrams, capable of simultaneously providing micro-deformation, FSS and compressive stress environments (Figure 5C). *In vivo* experiments demonstrated that this irregular scaffold structure optimizes fluid behavior, enhances osteoinductive properties and better matches the mechanical demands of bone regeneration, significantly improving osteogenic capacity. Building on this, researchers have explored integrating magnetic responsiveness into bone regeneration materials. Guo *et al*. [127] developed a magneto-mechano-mineralized scaffold combining a 3D-printed phosphorylated poly(glycerol sebacate) (PGS-P) framework with a hydrogel containing magnetic nanoparticles (MGel) (Figure 5D). Under an external magnetic field, this scaffold generated dynamic mechanical stimulation, activating the PIEZO1 signaling pathway and inducing β-catenin and YAP overexpression, thereby enhancing osteogenic differentiation and angiogenesis. Both *in vitro* and *in vivo* experiments confirmed excellent biocompatibility and bone defect repair ability of the scaffold, offering new strategies for bone tissue engineering. Similarly, Shao *et al*.’s [115] magnetically responsive mineralized collagen coating also validated the efficacy of magneto-mechanical stimulation in bone regeneration (Figure 5E). Using a rat calvarial defect model, they found that the MC + IOPs + Mag group significantly promoted new bone formation within 14 days, almost completely filling the defect beneath the implant. This work provides a new material design strategy for spatiotemporally controllable remote modulation of the bone microenvironment to promote osseointegration.

## Mechanical stimulation promotes muscle regeneration

As a highly organized tissue, the primary function of muscle is to generate force and drive motion [128]. This tissue comprises differentiated, mature, multinucleated and directionally aligned muscle fibers endowed with efficient contractile capabilities [129]. Empirical evidence demonstrates that mechanical stimulation guides cellular alignment along the axis of strain and substantially enhances muscle tissue growth and development [130–133]. For example, myoblasts were seeded on a porous collagen scaffold and subjected to continuous or cyclic uniaxial strain. Continuous mechanical strain significantly induced the expression of matrix metalloproteinase-2, thereby promoting muscle repair [134–136].

Further studies have revealed that both skeletal muscle and satellite cells are sensitive to mechanical loading, with their biological characteristics and functions being modulated accordingly [137, 138]. Satellite cells are a major component of the human muscle maintenance system and are frequently activated to replace any lost myofibers [135]. Tensile stimulation activates satellite cells while enhancing glucose uptake and recruiting immune cells [139, 140]. *In vitro* experiments confirm that cyclic mechanical stretching optimizes myoblast alignment and increases myofiber diameter [141]. For instance, Vandenburgh’s team utilized a cell-stretching device to apply dynamic stretching to embryonic skeletal muscle cells, extending the substrate to 400% of its original length. This dynamic loading not only promoted cell proliferation and directional alignment but also induced myoblast fusion into structurally robust and densely packed myotube networks, which were 2–4 times longer than those formed under static culture conditions [141]. These findings collectively underscore the substantial therapeutic potential of mechanical stimulation in muscle injury repair and regenerative medicine. Additionally, studies demonstrate that diaphragmatic mechanical stress activates the MAPK pathway through a direction-dependent bifurcation mechanism, with transverse stretching proving more efficient than longitudinal stretching at equivalent intensities in inducing downstream factor activity. The two signaling pathways diverge: the longitudinal route operates via the PI3K-Ca^2+^-PKC-MEK cascade, while the transverse pathway requires protein kinase A involvement to achieve distinct transcriptional regulation [138]. Among these, p38α MAPK serves as a critical mechanotransduction hub, regulating both skeletal muscle differentiation/myogenic fusion [142, 143] and activating quiescent satellite cells [144]. Separately, research by Kotaro Hirano has shown that PIEZO1, a mechanosensitive ion channel, which senses membrane tension and mediates Ca^2+^ influx to precisely control regeneration of muscle satellite cells [145].

Furthermore, massage therapy activates FAK and ERK1/2 pathways through mechanotransduction, triggering phosphorylated protein cascades that synergistically suppress inflammation and restore metabolic homeostasis [14]. Taken together, mechanical stimuli orchestrate multifaceted mechanisms including cellular alignment optimization, signal transduction modulation and metabolic adaptation regulation, playing a pivotal role in muscle development, regenerative repair and functional homeostasis maintenance.

Skeletal muscle, as a vital component of the human body, constitutes 40–45% of total body weight and plays an irreplaceable role in driving bodily movement and maintaining somatic morphology [146]. Despite possessing significant self-repair and regenerative capacity [147], the natural repair mechanisms of skeletal muscle falter in addressing tissue defects and functional impairments caused by chronic diseases or severe trauma [148]. Notably, when original tissue mass loss exceeds 20%, the intrinsic repair capability of muscle becomes insufficient to compensate for the defect, consequently leading to chronic functional deficits [149]. Current mainstream strategies for skeletal muscle repair focus on autografts, allografts and synthetic implants [137, 150]. Nevertheless, these methods suffer from multiple limitations including donor-site morbidity, scarcity of donor tissues, immune rejection and suboptimal postoperative rehabilitation outcomes [151, 152]. Against this backdrop, mechanical stimulation emerges as an intervention measure capable of effectively promoting muscle regeneration and repair, thereby opening up a more promising therapeutic pathway for skeletal muscle injury restoration.

**Figure 6:**
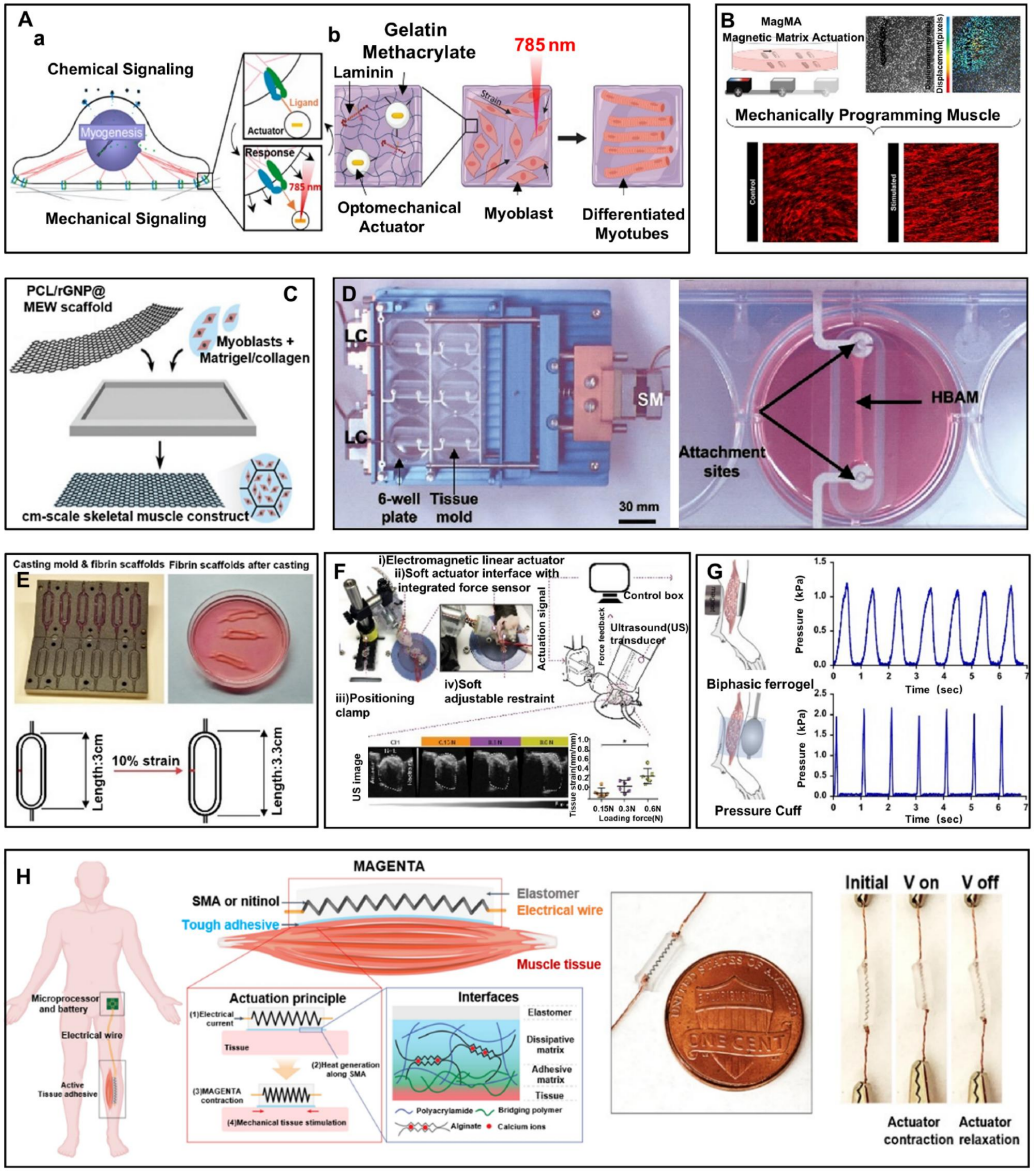
Mechanical stimulation devices used for muscle regeneration and repair. (**A**) (**a**) Mechanical stimulation based on photoresponsive nanoparticle actuators to promote myogenesis. Reproduced from Ref. [153] with permission of American Chemical Society, © 2020. (**b**) An optomechanical-driven hydrogel platform for mechanical stimulation with spatial and temporal resolution. Reproduced from Ref. [154] with permission of American Chemical Society, © 2023. (**B**) A method of MagMA that controls the direction and velocity of permanent magnets to drive actuation for mechanical stimulation. Reproduced from Ref. [155] with permission of Cell Press, © 2023. (**C**) A 3D matrigel/collagen/MEW scaffold-based magnetic deformation platform for dynamic skeletal muscle culture. Reproduced from Ref. [156] with permission of Wiley-VCH, © 2023. (**D**) A MCS4 for improving tissue-engineered human skeletal muscle. Reproduced from Ref. [157] with permission of American Physiological Society, © 2002. (**E**) A MagneTissue for rapid engineering of skeletal-muscle-like constructs. Reproduced from Ref. [158] with permission of Elsevier, © 2015. (**F**) An electromagnetic linear actuator integrated with a force sensor for promoting skeletal muscle regeneration. [159] (**G**) Biphasic ferrogel scaffolds implanted at the site of muscle injury to promote muscle regeneration. Reproduced from Ref. [160] with permission of National Academy of Sciences, © 2016. **(H**) MAGENTA, when implanted into the mouse leg to provide mechanical stimulation to the muscle, can prevent muscle atrophy. Reproduced from Ref. [20] with permission of Springer Nature, © 2022.

To mimic the dynamic mechanical microenvironment *in vivo* within static culture systems, photo-driven thermoresponsive materials offer a promising noninvasive strategy for applying mechanical stimulation. For example, Ramey-Ward *et al*. [153] reported the use of optical-mechanical actuator (OMA) particles (Figure 6A). The particle takes a gold nanorod as the core, encapsulated by a thermosensitive polymer hydrogel shell on the outer layer, and its surface is covalently modified with Arginine-Glycine-Aspartate (RGD) peptides that can specifically bind to integrin receptors on the surface of C2C12 myoblasts. When irradiated with near-infrared (NIR), the gold nanorod efficiently absorbs light energy and converts it into localized thermal energy through the surface plasmon resonance effect, rapidly raising the temperature of the hydrogel shell above its lower critical solution temperature and triggering instantaneous dehydration and shrinkage of the hydrogel; upon cessation of light irradiation, the hydrogel rapidly re-swells and expands. This periodic volumetric change generates periodic mechanical stimulation. By virtue of the specific binding between RGD peptides and integrin receptors, the periodic contractile force of the particle is directly transmitted to C2C12 myoblast receptors, thereby inducing directional cell elongation and promoting the nuclear accumulation of the transcription factor YAP1. Furthermore, while cells exist in mechanically dynamic environments *in vivo*, most *in vitro* cultures rely on static substrates such as plastic dishes or gels. To address this limitation, Ramey-Ward *et al*. [154] incorporated previously characterized OMA-actuated nanoparticle elements into a hydrogel-based cell culture substrate GelMA (Figure 6A), enabling the generation of periodic deformations under NIR illumination. This mimicked the repetitive dynamic strains that cells undergo in their *in vivo* environment, thereby providing a dynamic mechanical stimulation environment for the cells. The hydrogel-mediated mechanical stimulation enhanced myogenesis in C2C12 myoblasts and rescued differentiation in chronic inflammation models.

For the purpose of delivering dynamic and wireless mechanical stimulation to tissues, Rios *et al*. [155] developed a magnetic matrix actuation (MagMA) (Figure 6B). This method achieves synchronization of the alignment of skeletal muscle fibers and downstream functional muscle contractions, thereby enabling precise control over muscle morphology and force generation.

In addition, Cerardo-Servin *et al*. [156] employed melt electrowriting (MEW) technology to fabricate a magnetoactive microfiber network with a controllable hexagonal architecture (Figure 6C). This system utilized magnetized polycaprolactone as the composite matrix, achieving uniform dispersion of magnetic particles through *in situ* deposition of iron oxide nanoparticles onto graphene oxide surfaces. The material exhibited exceptional magnetic responsiveness, enabling out-of-plane reversible deformation under external magnetic fields below 300 mT. This property effectively guided C2C12 myoblast alignment along the microfiber orientation and facilitated the ordered formation of 3D myotube structures.

Furthermore, Powell *et al*. [157] designed and constructed a mechanical cell stimulator device, version 4 (MCS4; Figure 6D) to incorporate mechanical loading into 3D tissue engineering processes. The MCS4 was developed to simulate the repetitive muscle loading experienced during *in vivo* long bone growth and movement, such as the conditions observed during embryonic development, by applying cyclic stretching and relaxation cycles to human bioartificial muscles (HBAM) over an 8-day period. This intervention significantly enhanced the elastic properties of the constructs, achieving a 2- to 3-fold increase, while simultaneously promoting a 12% expansion in mean muscle fiber diameter and a 40% rise in the cross-sectional area occupied by muscle fibers.

To further optimize muscle engineering constructs, Heher *et al*. [158] developed a novel bioreactor system (MagneTissue) (Figure 6E), which applies mechanical strain to cells embedded in a fibrin matrix via magnetic actuation. The results showed that, upon strain application, structural genes critical for muscle functionality and contractility were significantly upregulated.

Furthermore, Seo and colleagues [159] developed a robotic device equipped with real-time force control, which possesses the capability to operate collaboratively with ultrasound imaging technology (Figure 6F). The specific implementation method involves using an electromagnetic linear actuator to generate driving force, measuring and feedback controlling periodic compressive loads through a force sensor. With a frequency of 1 Hz, three different forces (0.15 N, 0.3 N or 0.6 N) were applied to the mouse tibialis anterior muscle, and mechanical stimulation was continuously conducted for 14 days. Research has found that these three forces significantly reduced interstitial fibrosis and damaged muscle fibers, and there was no significant difference among the three force conditions. Notably, this method can rapidly deplete neutrophil populations and their mediated related factors, ultimately enabling severe muscle injury mouse models to exhibit significantly enhanced motor functional recovery effects. To enable force stimulation for promoting muscle injury repair *in vivo*, Cezar *et al*. [160] developed a biphasic iron hydrogel scaffold (Figure 6G). After implanting it into the injured site, they operated on the scaffold using a magnetic field to apply stimulation every 12 h for 5 min at a frequency of 1 Hz, thereby generating uniform cyclic compressive stress. The intervention effectively suppressed fibrous capsule formation around the implant and substantially mitigated fibrotic progression and inflammatory responses within the damaged muscle tissue. Meanwhile, controls such as pressure cuffs were set up. Both the biphasic iron hydrogel and the pressure cuff could exert uniform cyclic compression on the injured muscle, with similar forces applied. At 2 weeks, the specific peak isometric contraction force of the treatment groups stimulated by the biphasic iron hydrogel and the pressure cuff increased by 2.6 times and 2.2 times, respectively, compared to the untreated control group. Notably, the mechanical stimulation exerted by the biphasic iron hydrogel dramatically enhanced muscle regeneration compared to an untreated control group. The treatment cohort displayed a 2.3-fold increase in maximum contractile force at 2 weeks post-intervention. Although mechanical stimulation has been demonstrated to regulate diverse biological processes at cellular and tissue levels, its application in tissue regeneration and rehabilitation medicine remains constrained by limitations in compatible devices. Nam *et al*. [20] developed a gel-elastomer-nitinol composite tissue adhesive with a mechanically active gel-elastomer-nitinol tissue adhesive (MAGENTA) to promote the muscle regeneration (Figure 6H). MAGENTA comprises shape memory alloy springs capable of precise actuation under strains up to 40%, coupled with an adhesive component that efficiently transmits mechanical forces to deep tissues. This system generates and delivers muscle contraction-mimicking stimuli to target tissues with tunable intensity and frequency. The results show that MAGENTA can prevent and treat muscle atrophy while promoting muscle regeneration.

## Mechanical stimulation promotes repair of other tissues

**Figure 7:**
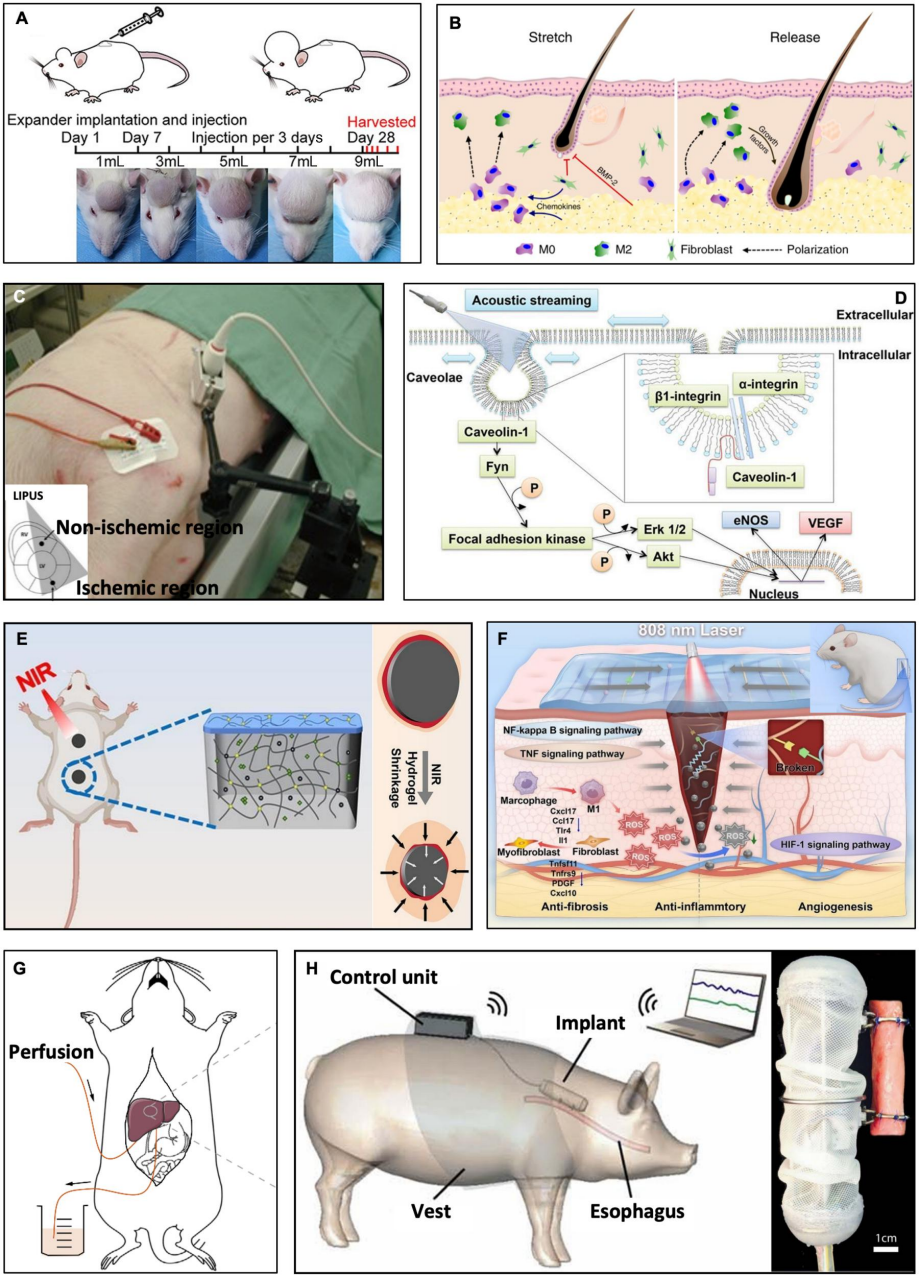
Research on the effects of mechanical stimulation in other tissue regeneration. (**A**) Rat scalp tissue expansion model. Expansion was performed every 3 days, and 1 mL of sterile saline was injected into the expander each time. Expanded skins were collected at 6, 12, 24, 48 and 72 h after the last expansion. Reproduced from Ref. [164] with permission of Oxford University Press, © 2024. (**B**) Schematic of the molecular basis of stretch-induced hair regeneration. Reproduced from Ref. [165] with permission of Springer Nature, © 2019. (**C**) LIPUS therapy in a porcine model and schematic images of LIPUS application. Reproduced from Ref. [60] with permission of PLOS, ©2014. (**D**) Schematic diagram of the mechanism underlying LIPUS-induced angiogenesis. Reproduced from Ref. [166] with permission of American Heart Association, ©2016. (**E**) *In vivo* assessment of D-NM3P1 hydrogel efficacy in wound healing applications. Reproduced from Ref. [167] with permission of Elsevier, © 2025. (**F**) a multifunctional bilayer hydrogel patch with photothermal-driven directional contraction and bioregulation for enhanced wound healing. Reproduced from Ref. [168] with permission of Elsevier, © 2025. (**G**) An *ex vivo* mouse liver perfusion model that promotes liver regeneration and repair through shear stress. Reproduced from Ref. [169] with permission of Elsevier, © 2023. (**H**) The usage diagram and structure of the robotic implant device. Reproduced from Ref. [170] with permission of American Association for the Advancement of Science, © 2025.

In addition to promoting repair of nerves, bone and muscles, mechanical stimulation has broad potential in other tissue regeneration and functional recovery [161–163]. For instance, Du *et al*. [164] reported a scalp expansion model by implanting silicone tissue expanders under the scalps of Sprague-Dawley (SD) rats for skin regeneration (Figure 7A), and applied expansion force through injecting normal saline. The experimental results showed that mechanical stretch could induce skin autophagy, which reached its peak at 48 h after expansion. Activation of autophagy with rapamycin significantly increased the expanded skin’s area and thickness, promoted cell proliferation and stem cell numbers, reduced apoptosis, improved angiogenesis and collagen synthesis, and thus, accelerated skin regeneration; conversely, autophagy inhibition diminished these effects and increased necrosis risk. Similarly, mechanical stretch has shown benefits in hair regeneration. Chu *et al*. [165] found that moderate mechanical stretching (33% strain for 7 days) induced macrophage M2 polarization, which released growth factors such as hepatocyte growth factor (HGF) and insulin-like growth factor-1 (IGF-1), activating hair follicle stem cells and promoting hair regeneration. This process depended on chemokine-mediated macrophage recruitment and functional transformation (Figure 7B).

In addition, low-intensity pulsed ultrasound (LIPUS), as a form of noninvasive mechanical force stimulation, has also achieved significant progress in the field of cardiovascular regeneration. Emanueli *et al*. [60] established a chronic myocardial ischemia model in domestic pigs by implanting Ameroid constrictors (Figure 7C). After 4 weeks of treatment in the experimental group (193 mW/cm^2^, 32 cycles, 20 min per session, 3 times a week), the left ventricular ejection fraction, capillary density in the ischemic region and regional myocardial blood flow were significantly increased, and the protein expressions of VEGF, endothelial nitric oxide synthase (eNOS) and basic fibroblast growth factor (bFGF) were up-regulated, while no obvious changes were observed in the sham operation group. Furthermore, Shindo *et al*. [166] using an acute myocardial infarction mouse model, further revealed that LIPUS improved cardiac function and reduced mortality by promoting angiogenesis, upregulating VEGF and eNOS and relying on caveolin-1 and β1-integrin-mediated mechanotransduction (Figure 7D). These results suggest that low-intensity pulsed ultrasound can effectively drive the regenerative repair process of ischemic myocardial tissue by regulating key molecular pathways, which provides a promising noninvasive therapeutic approach for ischemic heart disease.

Additionally, Tian *et al*. [167] developed a thermoresponsive contractile anisotropic nanocomposite hydrogel dressing that generates centripetal contraction force under thermal stimulation. The hydrogel is primarily constructed with a thermosensitive poly(N-isopropylacrylamide) matrix and polydopamine nanoparticles. Upon irradiation with an 808 nm NIR laser, the hydrogel dressing can rapidly elevate temperature and undergo contraction. The mechanical stimulation generated by this contraction upregulates the expression of integrin β1, which in turn strengthens the interactions between cells and the extracellular matrix. Meanwhile, it facilitates the polarization of macrophages from the pro-inflammatory M1 phenotype to the pro-regenerative M2 phenotype, thus accelerating the resolution of inflammation and promoting tissue regeneration (Figure 7E) [167]. In a full-thickness skin defect model of rats, the group treated with the hydrogel combined with NIR irradiation achieved a wound closure rate of over 50% within merely 3 days. Similarly, Ma *et al*. [168] fabricated a bilayer hydrogel patch capable of photothermal-driven directional contraction. In incisional injuries on the backs of rats, this patch exhibited excellent skin wound repair capabilities (Figure 7F). Li *et al*. [169] established an *ex vivo* mouse liver perfusion model, where the liver was connected to a Krebs–Henseleit buffer perfusion system via portal vein cannulation to simulate the physiological blood flow state and the blood flow state after hepatectomy. In the study, two perfusion conditions (4 mL/min and 8 mL/min) were set up, and it was found that high-flow-rate perfusion enhanced the interstitial flow in the space of Disse. This indicates that FSS exerts a direct promoting effect on liver repair without impairing the viability of hepatocytes (Figure 7G).

Moreover, Damian *et al*. [170] developed an implantable robotic device (Figure 7H), whose core structure includes open stainless steel attachment rings, a worm gear drive system and is encapsulated with biocompatible silicone rubber to isolate bodily fluids. The researchers established an esophageal regeneration model in female Yorkshire pigs through surgical procedures. On the morning of the second day after surgery, the device applied an initial traction force of approximately 2 N to the esophageal tissue to initiate the mechanical force stimulation process, and subsequently maintained continuous stimulation by increasing the ring spacing by 2.5 mm daily. The experimental results showed that the zero-strain length (the actual length of the tissue when it returns to a tension-free state after the residual force and strain on the tissue are removed) of the stretched esophageal segment in the surgical group increased significantly, while the esophageal lumen diameter and muscular layer thickness remained at normal levels throughout the experiment, with no tissue damage caused by traction. This result indicates that the precise, continuous and controllable traction force applied by the robotic device can effectively induce the tissue regeneration of tubular organs (such as the esophagus) without impairing their original functions, providing a new mechanism of action and technical solution for the treatment of tubular organ-related diseases such as long-gap esophageal atresia and short bowel syndrome.

## Conclusion and outlook

This review focuses on the advances of materials and devices of mechanical stimulation in the fields of injured nerve, skeletal muscle and bone injury regeneration. To date, researchers have successfully developed various materials and devices for delivering mechanical forces to cells [23, 171]. However, existing devices generally suffer from complex structures, which not only limits their widespread adoption as standardized laboratory tools but also hinders the effective translation of findings from *in vitro* experiments to *in vivo* applications [4, 88, 172–174]. To simplify the strategies, implementation of mechanical force stimulation through noninvasive approaches such as magnetic, light and ultrasound has been adopted, offering new directions to overcome the aforementioned bulky tools. Although some studies have achieved phased results in animal models, the direct clinical application of similar protocols remains challenging due to the complexity of human physiological environments [175–180].

Based on how cells perceive external stimuli, mechanical stimulation can be classified into three primary categories: modulating the stiffness of cell culture substrates, engineering surface topography of the substrate, and directly applying external mechanical forces to cells [181, 182]. Among the implementation methods of mechanical stimulation, directly applying mechanical forces to tissues represents the most intuitive intervention approach. Such methods utilize external force loading devices or bioreactor systems to precisely regulate the magnitude and frequency of stress exerted on target tissues. Although this strategy has demonstrated potent capabilities in modulating cell fate during *in vitro* studies, its clinical translation faces substantial challenges [88]. Future efforts should prioritize breakthroughs in developing miniaturized, intelligent mechanical loading devices, alongside establishing dynamic mechanical models that align with human physiological characteristics, to advance this technology toward clinical application [183]. For surface topography strategy, achieving precise regulation of morphology distribution at the microscale/nanoscale within 3D spaces remains a significant technical challenge. Future efforts should focus on deciphering the interplay mechanisms among distinct topographic features and developing technologies capable of exact spatial pattern control in three dimensions. Despite variations in the underlying mechanisms through which these mechanical signals are detected by cells, they collectively demonstrate regulatory effects on cellular behaviors and exert profound influences on the composition and architecture of the ECM [39]. Nevertheless, elucidating the intricate relationships among macroscopic mechanical forces, substrate stiffness, microscale/nanoscale topographic cues and corresponding cellular molecular responses remains a critical challenge.

To overcome the constraints of invasive procedures, remote manipulation systems based on magnetic materials, light-responsive materials and ultrasound-responsive materials [184]. While these approaches overcome spatial constraints inherent in *in vitro* models, it confronts numerous engineering challenges, including maintaining long-term stability of biocompatible materials and managing foreign body responses elicited by implanted devices, as well as the nondegradability requiring a secondary surgical procedure for removal. Additionally, the magnetic strategies are limited by their poor manipulate resolution. Light-responsive material systems suffer from limited tissue penetration depth and susceptibility to interference from biological tissue absorption. Ultrasound-responsive material systems face the challenge that their targeting accuracy is prone to being affected by the *in vivo* acoustic environment. These factors all render the practical application of the relevant technologies quite challenging. Although substantial progress has been made in developing methodologies for delivering mechanical stimuli, continued advancements are required to enhance both efficiency and precision. Such progress would substantially enhance the ability of strategies to orchestrate biological responses in biomaterials, thereby accelerating the development and application of intelligent, tissue-inductive biomaterials. Future research priorities are expected to shift toward comprehensive elucidation of the spatiotemporal regulatory mechanisms underlying mechanical signaling and cell fate determination, thereby providing more informative guidance for designing smart biomaterials tailored for regenerative medicine. The advantages and limitations of varying mechanical stimulation strategies based on loading devices or materials, operation mechanisms and applied tissues are summarized in Table 1.

**Table 1:** Advantages and limitations of varying mechanical stimulation strategies based on loading devices or materials, operation mechanisms and applied tissues.

Mechanical stimulation strategies		Main devices/materials	Operation mechanism	Applied tissues	Advantages	Limitations	Reference
Direct mechanical stimulation	Stretching	Neural prostheses, Silicone tissue expanders, Elastic polymer membrane, Distractor, PDMS substrate, GelMA-based materials, MCS4, MAGENTA	Cyclic stretching, continuous tensile force	Nerve Bone Muscle Skin Hair Tubular organs			[18, 20, 52, 81–84, 112–114, 124, 125, 157, 164, 165, 170]
	Compressive stress	Porous scaffold, Electromagnetic linear actuator, Biphasic iron hydrogel scaffold	Cyclic compression, static pressure	Bone Muscle	High bionic degree	Invasive and bulky, high risk of infection, complex operation, limiting clinical translation	[126, 159, 160]
	Fluid Shear stress	HA-on-chip, Fluid shear-stress loading device, Perfusion-flow bioreactor, Porous scaffold, Buffer perfusion system	Fluid shear stress, perfusion flow, interstitial flow	Bone Liver			[109–111, 126, 169]
Indirect mechanical stimulation	Photothermal	OMA, Nanocomposite anisotropic hydrogel, Bilayer hydrogel patch	NIR light triggers thermo-mechanical conversion to generate a periodic mechanical force	Muscle Skin	Noninvasive, precise targeted stimulation	Limited tissue penetration depth	[153, 154, 167, 168]
	Ultrasound	Ameroid constrictors, Transducer	Ultrasonic mechanical waves induce the acoustic streaming effect and cell membrane vibration	Cardiovascular	Deep penetration depth, noninvasive	Limited targeting accuracy	[60, 166]
	Magnetic	Magnetic hyaluronic acid hydrogel, Microfiber network, Bioreactor system, Mineralized collagen coating, Nanocomposite scaffold, hMMA nanoparticles, SPIONs, MagMA, MMP-SA,	Driven by external magnetic field, magneto-mechanical conversion generates microforces	Nerve Bone Muscle	Simple device design, noninvasive, deep penetration depth	Potential toxicity of nanoparticles	[57–59, 85, 86, 93, 115, 127, 155, 156, 158]

At the material and structural design level, it is necessary to consider the correlation between material composition, microstructure, mechanical properties and repair efficacy [185–189]. As the composition and microstructure of biomaterials directly affects their mechanical properties [64, 190–193], the formulation and optimization of material in the design process are of critical importance [194, 195]. For biodegradable devices and materials, it is essential to align the kinetics of material degradation with the required mechanical performance and the tissue repair cycle [11, 196–199].

At the level of basic mechanism research, it is essential to focus on the correlation between mechanical stimulation, cellular response and tissue repair, so as to provide a core basis for the technical optimization of materials and devices related to mechanical stimulation [200–202]. Currently, there remain numerous unknowns regarding the cellular sensing mechanisms corresponding to different mechanical stimulation modes, especially the regulatory rules of intracellular signaling networks under the synergistic effect of multidimensional mechanical signals have not yet been clarified. In the future, technologies such as single-cell sequencing and *in vivo* imaging can be employed to track the entire process of mechanical signal transmission from the material surface to the interior of cells [203]. Emphasis should be placed on analyzing the relationship between mechanical parameters and cell proliferation and differentiation, clarifying the differences in signal transduction corresponding to different mechanical stimulation modes and achieving precise and personalized regulation of mechanical stimulation for the loading devices and materials.

Furthermore, at the technical research and development level, it is imperative to drive the miniaturization, intelligence and noninvasiveness of mechanical stimulation devices [204, 205]. On one hand, efforts should be made to address the issue of complex structures and poor clinical adaptability of direct external force stimulation devices. On the other hand, to mitigate the inherent drawbacks of remote manipulation strategies such as magnetic, optical and ultrasonic approaches, materials optimization such as improving the photosensitivity for optical method and enhancing the resolution for magnetic and ultrasonic method is required. Moreover, leveraging the synergy of multiple stimulus-responsive modes, which harnesses the benefits of different noninvasive stimuli, represents a critical direction for enhancing tissue regeneration.

Overall, research on mechanical stimulation materials and devices in the field of tissue injury repair has made significant progress, with noninvasiveness and remote control emerging as key development trends. However, their efficiency and precision still need continuous improvement. To further improve tissue repair outcomes, future efforts should be directed toward correlating material properties with mechanical properties and clinical outcomes, understanding the intracellular mechanisms of mechanotransduction and integrating synergistic strategies.
